# Barcode Server: A Visualization-Based Genome Analysis System

**DOI:** 10.1371/journal.pone.0056726

**Published:** 2013-02-15

**Authors:** Fenglou Mao, Victor Olman, Yan Wang, Ying Xu

**Affiliations:** 1 Computational Systems Biology Lab, Department of Biochemistry and Molecular Biology and Institute of Bioinformatics, University of Georgia, Athens, Georgia, United States of America; 2 BioEnergy Science Center (BESC), University of Georgia, Athens, Georgia, United States of America; 3 College of Computer Science and Technology, Jilin University, Changchun, China; Queen's University Belfast, United Kingdom

## Abstract

We have previously developed a computational method for representing a genome as a *barcode* image, which makes various genomic features visually apparent. We have demonstrated that this visual capability has made some challenging genome analysis problems relatively easy to solve. We have applied this capability to a number of challenging problems, including (a) identification of horizontally transferred genes, (b) identification of genomic islands with special properties and (c) binning of metagenomic sequences, and achieved highly encouraging results. These application results inspired us to develop this barcode-based genome analysis server for public service, which supports the following capabilities: (a) calculation of the k-mer based barcode image for a provided DNA sequence; (b) detection of sequence fragments in a given genome with distinct barcodes from those of the majority of the genome, (c) clustering of provided DNA sequences into groups having similar barcodes; and (d) homology-based search using Blast against a genome database for any selected genomic regions deemed to have interesting barcodes. The barcode server provides a job management capability, allowing processing of a large number of analysis jobs for barcode-based comparative genome analyses. The barcode server is accessible at http://csbl1.bmb.uga.edu/Barcode.

## Introduction

Genome analyses, particularly comparative genome analyses, have been mostly driven by efforts to study specific scientific questions or to validate specific hypotheses about individual genomes. Discovery-driven studies of genomes and comparative genomes have been difficult to do due to the non-intuitive nature of the genomic sequence data although enormous amount of information is known to be encoded in such data, which could potentially be discovered more easily if the genomes were represented in a more intuitive manner. Here, we present one such intuitive representation of genomes, called *genome barcodes* based on our previous work [1], which is information-rich, intuitive and preserves various key properties of the distinct genomic regions.

The barcode representation is, in essence, a 2-dimensional grey-level image with the x-axis presenting all k-mers (or k-mers combined with their corresponding reverse complements) arranged in the lexicographic order, and the y-axis being the genome axis condensed by W fold, where the whole genome is partitioned into a sequence of windows (fragments) of W base-pairs (bps); each entry of the 2-D array is the frequency of a specific k-mer (or k-mer/reverse complement) inside the corresponding window, which is then mapped to a grey level in a genome-independent way so that higher frequencies are mapped to brighter grey levels [1]. An interesting property of this representation is that the majority of fragments generally have stable frequencies across the whole genome, hence giving rise to a vertical line for each k-mer with generally consistent grey levels for the majority portion of the genome (see Figure S1 for example) and collectively forming a barcode-like image, hence the name. We have previously made a number of observations about a few interesting properties of the so-defined barcode representation of a bacterial genome [1]: (a) the majority of the sequence fragments of a bacterial genome, on average 87–88%, has highly similar barcodes; (b) a substantial portion of the genomic regions with distinct barcodes from the rest of a bacterial genome is found to be related to bacteriophages and other transferred material; (c) barcode-based similarities, at a genome scale, correlate well with genome sequence similarities when sequence similarities are relatively high (at least 65%); and (d) genomes of different kingdoms, such as prokaryotic, eukaryotic, and even mitochondrial and plasmid genomes, share common barcode properties distinguishing them from those of the other kingdoms. While our previous studies have been mostly focused on bacterial genomes, we notice that eukaryotic genomes tend to have richer and more complex barcode properties, making them more interesting to visualize and to discover interesting barcode-based patterns, conserved or unique, across different types of genomic regions and different genomes, and further associate them to well understood biological functions.

One highly promising application area for barcodes is in inference of horizontally transferred genes (HTGs). Compared to the existing composition-based HGT detection tools such as the method developed by Garcia-Vallvé *et al.* [2] and *Wn* developed by Tsirigos *et al.* [3], barcode-based analysis tends to be more sensitive and accurate based on our preliminary studies since it does not just use individual k-mer frequencies but instead the information about all the k-mer frequencies as a whole, manifested through the texture of a barcode image [1]. Another interesting application area is in analyses of the rapidly increasing metagenome sequences, such as metagenome binning for separation of genomic fragments into prokaryotic, eukaryotic and virus sequences, into different taxonomy groups, and into chromosomal *versus* plasmids sequences, depending the needs.

Various other applications of genome barcodes are currently being carried out in our lab, focused on, for example, (i) prediction of the relative residential time among predicted horizontally transferred genes in a host genome, (ii) classification of repetitive elements in human and plant genomes, (iii) identification of viral islands in bacterial genomes among other things, and (iv) systematic analyses of genomic regions in the human genome with unusual barcodes. In a sense, genome barcodes provide an effective tool for linking related genomic sequences with similar barcode images, which extends sequence homology-based genome analysis in such a way that related genomes tend to have similar barcodes regardless of whether they have sequence-level similarities or not – it represents another level of similarity at a more coarse scale but also a more fundamental level than sequence comparison, which has not been systematically used in genome analyses.

To facilitate barcode-based genome and comparative genome analyses, we have developed a working environment, *the barcode server*, which supports a suite of barcode-based analysis and visualization tools. We believe that this capability will allow genome researchers and graduate/undergraduate students to mine genomes using the tools already provided in the barcode sever and additional new tools to be added.

## Algorithms and Implementation

The barcode server currently supports three key capabilities, namely (a) generation of barcode image(s) for provided DNA sequence(s), (b) detection of sequence fragments in provided sequence(s) with abnormal barcodes compared to the rest of the sequences, and (c) clustering of the provided sequence fragments into groups with similar barcodes.

### 1. Barcode image generation

The algorithm for barcode generation for a given DNA sequence is given in [1]. We now outline the basic idea of the algorithm along with a few issues related to parameter setting. For a provided DNA sequence, the algorithm uses four parameters, the k-mer size, the window size W to calculate the barcode image of the DNA, a flag for calculating single or double stranded genome barcodes, and a flag for calculating frequencies of contiguous k-mers or k-mers with specified spacers. It first partitions the genome into non-overlapping and consecutive windows of the same size, i.e., W bps (possibly except for the last window with a smaller size). The algorithm can generate both single and double-stranded barcodes. For single-stranded barcode calculation, the algorithm calculates the frequency for each k-mer within each window of W bps; and for double-stranded barcode calculation, the algorithm treats each k-mer and its reverse complement as one single k-mer. The reason we consider these two cases separately is that single-stranded barcodes are effective in distinguishing among different types of genomic regions in the same genome while double-stranded barcodes are more effective in distinguishing among different genomes as we have previously discovered. We believe the reason is that the double-stranded barcodes, which have more k-mers and more stable than single stranded barcodes, are information-richer in representing a whole genome (or a whole DNA sequence) while single-stranded barcodes are more sensitive to specific classes of genomic regions such as coding regions, inter-genic and inter-operonic regions in bacteria, or repetitive elements in higher eukaryotes. A user can specify if the k-mer frequencies need to be calculated based on k consecutive or non-consecutive positions with specified spacer sizes. With the specified (or defaulted) parameter values, the algorithm maps the (calculated) frequencies to grey levels using the same mapping scheme for any genome, so the same k-mer frequencies across different genomes are mapped to the same grey levels; and hence barcodes for different genomes can be compared based on image similarities.

The current default parameters in the barcode server for bacterial genomes are k = 5, W = 3,000 bps, “double-stranded barcode” and “consecutive k-mers”, which can be adjusted by the user. The rule of thumb in adjusting the parameters is that for larger genomes, a user may want to use larger k as well as W values; and generally we advise the user to use k = 4 or 5. For metagenomic data, we typically suggest using k = 3 or 4 and W = 500. Set the flag to “double-stranded barcode” if the goal is do comparative genome analyses; otherwise to “single-stranded”.

If the input file is a Fasta file with multiple short sequences, the user does not need to specify the parameter for window size (W) as required for one single long DNA sequence. Since the input file contains sequence fragments, we don't need to cut them into pieces. Because the input sequences have different lengths, we used another parameter “reference size” to normalize its k-mer frequency. The basic idea for the normalization is to modify the k-mer frequency proportionally based on the total number of k-mers in the reference size.

### 2. Detection of sequence fragments with abnormal barcodes

The barcode server supports a capability for identification of genomic sequence fragments with distinct (abnormal) barcodes in comparison with the majority of a given sequence [1]. We provide two algorithms for this purpose. The first algorithm is a statistics-based method. The algorithm first partitions the a given sequence into a list of non-overlapping and consecutive fragments of the same size (the default size = 1,000 bps). For *k*-mer with index *j* we find the shortest interval [*a_j_*,*b_j_*] containing at least *X*% (*X* is a user-specified parameter) of *j*-th *k*-mer frequencies calculated for all fragments. For *i*-th fragment we define the value *F(i)* as a number of *k*-mers with frequencies for the fragment that got outside of corresponding interval [*a_j_*,*b_j_*]. It's clear that the higher the value *F(i)* the more “non-native” the *i*-th fragment is. From practical point of view *F(i)* is binomial random variable *Bi*(*K*,0.01*X), where *K* is a number of *k*-mers. The algorithm sorts values *F(i)* in increasing order, and as a threshold we choose *i_0_* that provides the maximal value for *F(i+1)+F(i−1)−2F(i)* (the approximation of a second derivative for sorted values of *F(i)*). We consider all fragments *i* with *F(i)> = F(i_0_)* to be the non-native fragments of the given sequence. We noticed that the abnormal fragment prediction is not very sensitive to the detailed value of *X* within the range from 5 to 20. So we have chosen *X* = 10 as the default value of our program, which can be adjusted by the user.

The second algorithm is a distance based method. Euclidean distance between a vector of k-mers frequencies of each fragment and the average vector of frequencies is calculated for all fragments. The ones with large Euclidean distances from the average vector are considered as the ones with abnormal barcodes while the detailed distance cutoff can be specified by the user.

### 3. Barcode-based clustering of sequence fragments

Our clustering algorithm is a modification of Kruskal's algorithm [4] for building a minimum spanning tree over the given set of sequence fragments to be clustered. We define the distance between two sequence fragments as the Euclidean distance between the two vectors of their k-mer frequencies. Initially, each sequence fragment is defined as a separate cluster. We then merge two clusters with the shortest inter-cluster distance among all such distances into one cluster, where the distance between two clusters is defined as the average distance between each pair of sequence fragments, with one from each cluster, over all possible such pairs; we repeat this till exactly K clusters exist, where K is a user-specified number of clusters.

We found that this algorithm is both sensitive to identification of small sized clusters and highly stable in the presence of noises, and generally do not over-partition clusters. Generally the algorithm outperforms K-means clustering algorithm. The key reason is that while a K-means algorithm tends to generate clusters with similar sizes, our algorithm identifies clusters with varying sizes.

### 4. Implementation

**Figure 1:**
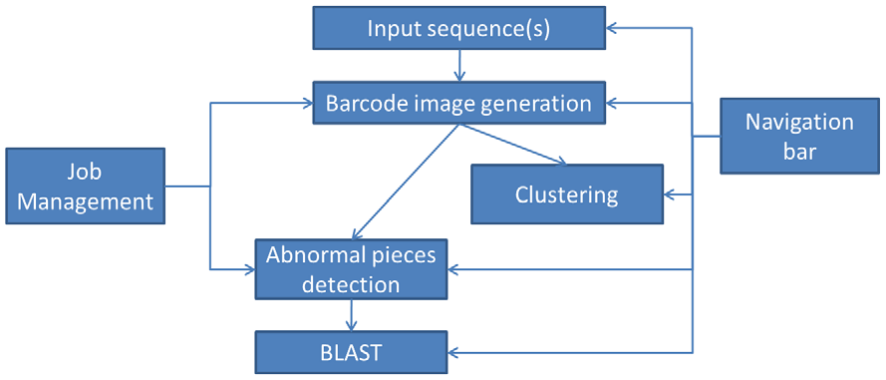
Webserver organization.

The barcode webserver provides services for generation of genome barcodes, detection of sequence fragments with abnormal barcodes and clustering of sequence fragments with similar barcodes for a given set of genomic sequences, using algorithms outlined in Section 2. The webserver also provides a navigation bar to guide a user to go through intermediate computational results, and a job management interface to examine all the finished jobs. The webserver structure is outlined in Figure 1.

The main component of the webserver has two computational pipelines. The first consists of the following steps: input sequence(s)→barcode image generation→detection of abnormal barcodes→sequence search using BLAST. This pipeline provides the capability of finding sequence fragments with abnormal barcodes and their potential origins. The second pipeline consists of: input sequence(s)→barcode image generation→clustering of sequence fragments based on their barcode similarities; this pipeline provides the capability of clustering sequence fragments into groups sharing similar barcodes. The navigation bar can guide the user to access previously saved result pages in the pipelines, and thus a user can specify different parameters; run the same computational job with different parameters multiple times; and compare the computed barcodes. The job management interface can be accessed from the front page of the server to access all the finished jobs and to do comparisons of different genome barcodes.

The server accepts input data from the user, and it also maintains a local sequence database for the user's convenience, currently consisting of all sequenced bacterial genomes, which supports up-loading sequence data from the database to the server. Currently the local database contains 1,562 DNA chains from 812 sequenced bacterial genomes.

In addition, the server supports a wiki page allowing users to post their requests for refinement of any existing functionalities of the server or for suggestions of adding additional capabilities. We will actively update the server system based on the suggestions from the users, and make any announcement of any key revisions through the wiki page.

The web server is implemented on a Linux Fedora 8 computer. Apache is used to host the server, and php is used to generate dynamic web pages. MySQL is used to manage the local data.

## Examples

We now provide three examples to show how to use the barcode server and to demonstrate its analysis capability.

### Example 1: identification of sequence fragments with foreign origins

We use *E. coli str. K-12 substr*. MG1655 as an example to illustrate how to identify sequence fragments in the genome possibly with foreign origins. The genome of *E. coli str. K-12* is stored in the local database of the server. We only need to click on the link “Start from the sequences in the server” on the front page to access a list of genomes stored in the local database. Then click on “*E. coli str. K-12 substr*. MG1655” to access the DNA chains of the organism. We can click on the chain “NC_000913” to access the barcode generation page. From there, we click on the “Submit” button to generate a barcode using the default parameters. The barcode image for the organism will be generated and displayed as shown in Figure S1. We then click on the “Detect abnormal pieces” link to open the page for detection of sequence fragments with foreign origins. After we click on the “Submit” button on this page, the server will start to identify sequence fragments with abnormal barcodes, again using the default parameters. Figure S2 shows such identified fragments, all marked using red lines, each of which points to a sequence fragment possibly with foreign origins. All the red lines are clickable. So we can click on one of them, e.g., the one with sequence coordinate 2100830–2109833 (Figure S2). From there, we can bring up the BLAST search page to search this sequence against the local sequence database, using the default e-value cutoff at 1e-6; and then click on the “Submit” button to find out the possible origin of this sequence fragment. From the returned Blast results, we found that this sequence has strong hits in *Bacilli*, suggesting the possible origin of this sequence.

### Example 2: clustering of metagenomic sequences

We now demonstrate the power of our clustering algorithm using a simulated metagenomic dataset, consisting of 2,500 non-overlapping sequence fragments of ∼500 bps long each, extracted from five bacterial genomes, namely *Escherichia coli str*. K-12 *substr.* MG1655, *Nostoc sp.* PCC 7120, *Synechococcus sp.* WH 8102, *Geobacillus kaustophilus* HTA426 and *Ureaplasma parvum serovar 3 str.* ATCC 27815. These fragments are mixed together and stored in a Fasta file [File S1].

We run the barcode server by clicking on the link “Start from a fasta file with multiple DNA sequences” to access the barcode generation page. Click on the button “Browse” to upload the sequence file. We then set “Reference size of sequence” to 500 and “Size of k-mer” to 4; then click on the “Submit” button to generate the barcode for the 2,500 sequence fragments. Then we click on the “Do a clustering” link to access the clustering page and to specify the clustering parameters. We set the desired number of clusters to 60 and the minimal cluster size to 10. We consider this many to-be-identified clusters because we know that the five bacterial genomes have many transferred genes with abnormal barcodes, and this high number will allow the user to see all the small clusters containing abnormal sequence fragments (we can consider a substantially small number of clusters, which will not affect the main clustering results). Then click on the “Submit” button. A table will appear to summarize the identified clusters. By clicking on the links under “Index” column, the fragments in the corresponding cluster will be shown. We can also visualize the clusters by clicking the links under “Barcode for the cluster” column [shown in Figure S3].

We used the following formula to assess the quality of the overall clustering result.
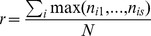
where s is the number of genomes (s = 5), *N* is the total number of fragments (N = 2,500), *i* goes through all the clusters of size >10, and *n_ij_* is the number of fragments in cluster *i* from genome *j*. Note that *r* will be 1 if the fragments in each cluster are from the same genome, and it will be less than 1 if the fragments from more than one genome are clustered together. The closer the *r* value is to zero, the poorer the clustering quality it represents. We obtained *r* = 0.84 for this test set, indicating that most of the fragments in each cluster are from the same genome. We checked the mis-clustered fragments, and found that the vast majority of them have abnormal barcodes compared to those of their host genomes. We repeated the same clustering after removing abnormal fragments from 5 genomes (using the first pipeline). The level of performance improved to *r* = 1.0.

### Example 3: search for pathogenic islands in *E. coli* O157

*E. coli* O157 is a pathogen while *E. coli* K12 is not. Considering that a pathogenic island is an inserted sequence from a phage, we would expect that it will have a distinct barcode pattern from that of the rest of the host genome.

**Figure 2:**
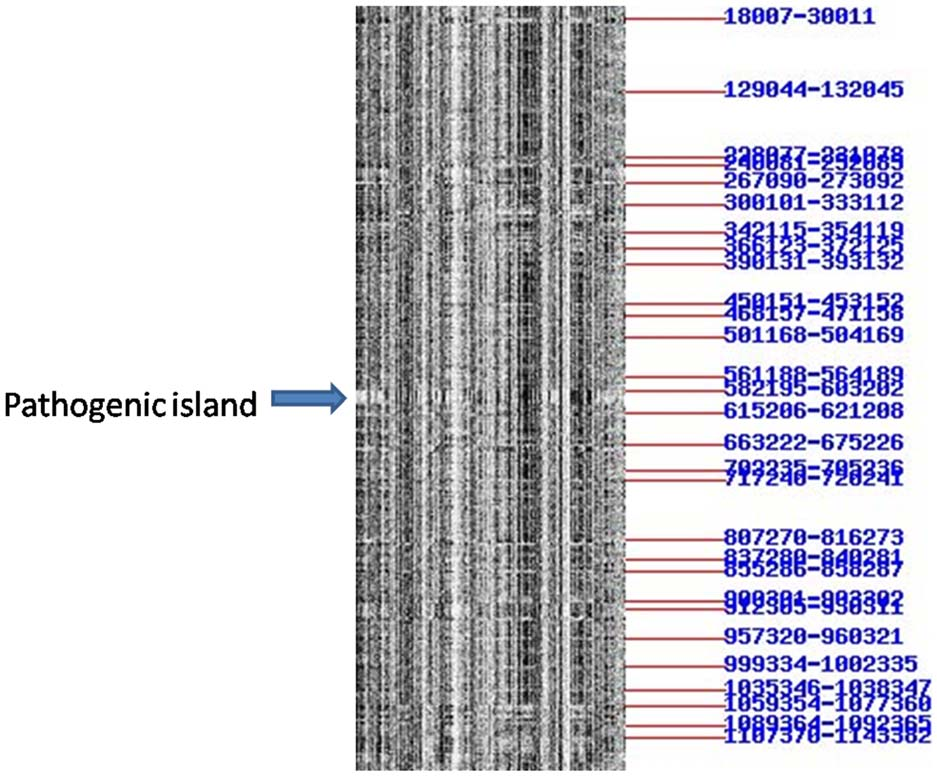
Barcode with detected abnormal pieces for *E. coli* O157:H7 EDL933.

Based on these criteria, we have visually and quickly examined the whole-genome barcodes of *E. coli* O157:H7 EDL933 [barcode shown in Figure 2] and *E. coli* K12 *substr.* MG1655 (we omit the detailed procedure here), to identify relatively long sequence fragments in *E. coli* O157:H7 EDL933 with distinct barcodes compared to the rest of *E. coli* O157:H7 EDL933 and the whole genome of *E. coli* K12 *substr.* MG1655. We quickly found one such fragment from sequence coordinate 582195 to 603202. We then run Blast search against the local bacterial sequence database, which found 18 hits, one being itself and three from other strings of *E. coli* O157; and none of the 18 are from *E. coli* K12. This study ultimately led to the confirmation of this sequence being a pathogenic island, as published in [5].

## Conclusions

We have developed the barcode server in support of genome and comparative genome analyses in an intuitive manner. Our preliminary study suggests that this visualization-based genome analysis tool can be used to solve a wide range of genome analysis and mining problems, particularly useful for making new discoveries about genomic sequences with interesting barcodes, either conserved across a group of genomes or unique to some special genomes or genomic regions. We will continue to add new utility tools in support of various genome and comparative genome analysis needs, as we find the new needs or our users suggest for new functionalities. With the continued expansion of the utility tools based on barcode representations of genomes, we believe the server will be a useful tool to the general genome research community, which will complement homology-based genomic analyses.

## Supporting Information

Figure S1 **The barcode for** ***E. coli str. K-12 substr.*** **MG1655.**
(TIF)

Figure S2 **Detected sequence fragments in** ***E. coli str. K-12 substr.* MG1655 with abnormal barcodes.**
(TIF)

Figure S3 **Barcode images for the major clusters.**
(TIF)

File S1 **Sample metagenomics fasta file.**
(FNA)

## References

[1] Zhou F, Olman V, Xu Y (2008) Barcodes for genomes and applications. BMC Bioinformatics 9: 546. 19091119 10.1186/1471-2105-9-546 PMC2621371

[2] Garcia-Vallve S, Romeu A, Palau J (2000) Horizontal gene transfer in bacterial and archaeal complete genomes. Genome Res 10: 1719–1725. 11076857 10.1101/gr.130000 PMC310969

[3] Tsirigos A, Rigoutsos I (2005) A new computational method for the detection of horizontal gene transfer events. Nucleic Acids Res 33: 922–933. 15716310 10.1093/nar/gki187 PMC549390

[4] Rosen KH, editor (1999) Handbook of Discrete and Combinatorial Mathematics. New York: CRC Press.

[5] Wang G, Zhou F, Olman V, Li F, Xu Y (2010) Prediction of pathogenicity islands in enterohemorrhagic Escherichia coli O157:H7 using genomic barcodes. FEBS Lett 584: 194–198. 19941858 10.1016/j.febslet.2009.11.067

