# Supplementary figures and images for: Barcode Server: A Visualization-Based Genome Analysis System
Source: PLoS One. 2013 Feb 15;8(2):e56726. doi: 10.1371/journal.pone.0056726 (PMC3574017; doi:10.1371/journal.pone.0056726)

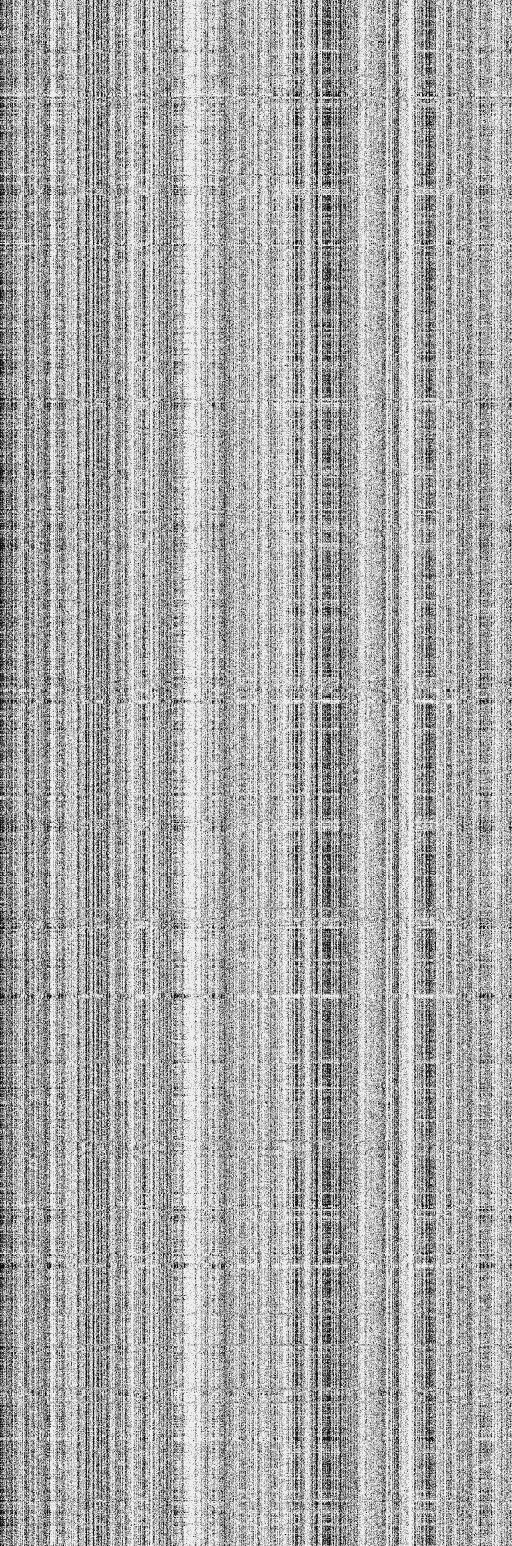

Supplement: Figure S1 — The barcode for E. coli str. K-12 substr. MG1655. (TIF) [file pone.0056726.s001.tif]

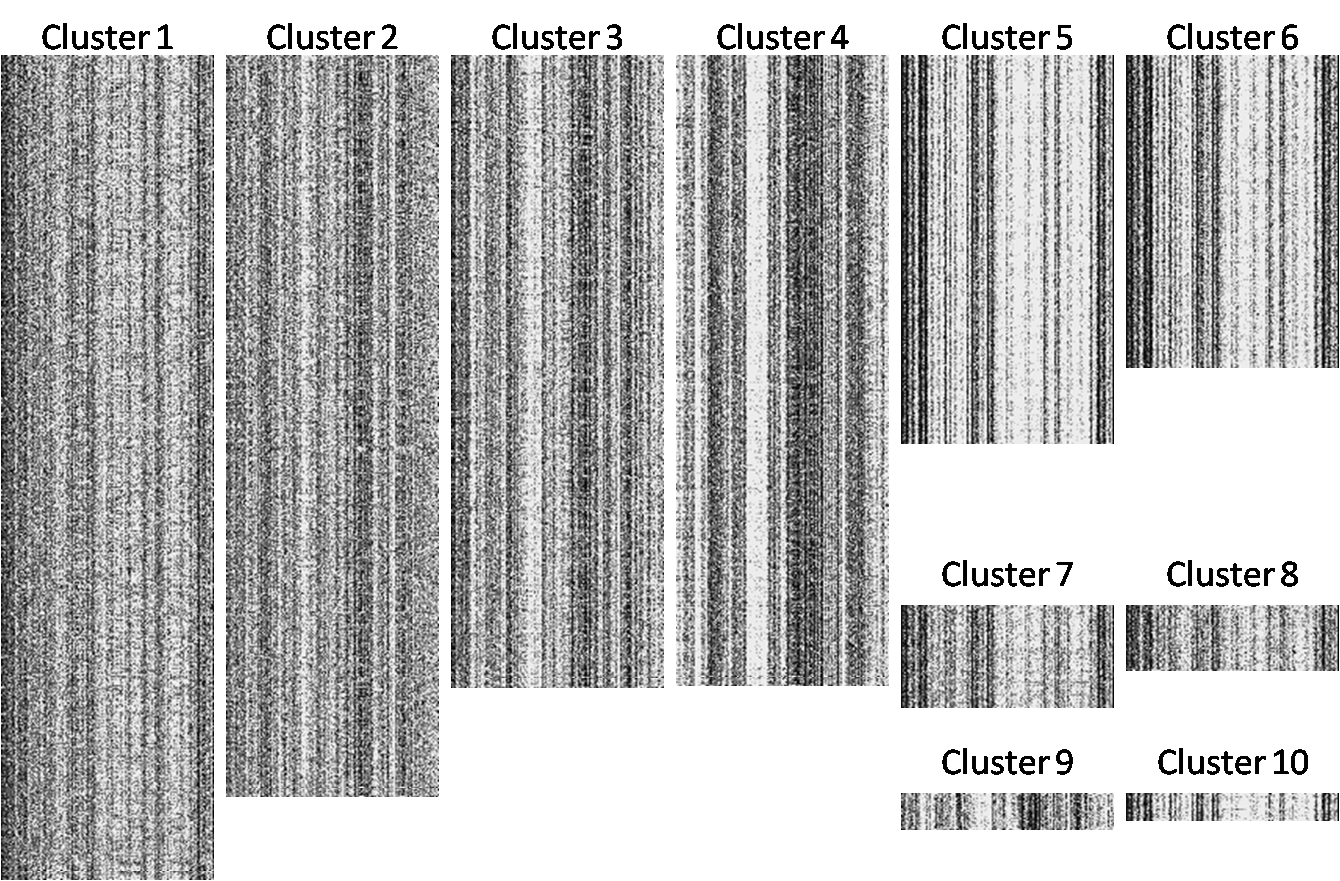

Supplement: Figure S3 — Barcode images for the major clusters. (TIF) [file pone.0056726.s003.tif]
